# A Nanopore-Only Assembly of a Nuclear and Mitochondrial Genome of a Red Coachwhip (*Masticophis flagellum piceus*)

**DOI:** 10.3390/genes17030307

**Published:** 2026-03-04

**Authors:** Alan F. Scott, David W. Mohr

**Affiliations:** Genetic Resources Core Facility, Department of Genetic Medicine, Johns Hopkins University School of Medicine, Baltimore, MD 21287, USA; dwmohr@jhmi.edu

**Keywords:** nanopore sequencing, snake, genome assembly, mitochondrial genome, field-based simulation

## Abstract

We report a chromosome-level assembly of a male red coachwhip snake (*Masticophis flagellum piceus*) generated exclusively with nanopore sequencing. Using Hifiasm-ONT for assembly and RagTag for scaffold polishing, we produced a 1.61 Gb nuclear genome comprising 8 macrochromosomes and 10 microchromosomes with a 97.7% BUSCO completeness score. Annotation with LiftOn found 19,832 loci, including 18,025 protein-coding genes. The mitochondrial genome, assembled with MitoHiFi and annotated with MitoFinder, was 17,119 bp with 13 coding genes, 22 tRNAs and 2 rRNAs. All sequencing was performed in a simulated mobile laboratory using a portable sequencer and a laptop with analyses done both locally and remotely. These results highlight the feasibility of decentralized genomics and its potential to accelerate biodiversity research globally.

## 1. Introduction

We are in an era of high-quality genomes because of many individual efforts and those of large consortia such as the Earth Genome Project (https://www.earthbiogenome.org/), the Vertebrate Genome Project (https://vertebrategenomesproject.org/) and the Darwin Tree of Life (https://www.darwintreeoflife.org/). This progress has been made possible largely by advances in long-read sequencing and improved software for DNA assembly and performed in large facilities with substantial equipment and informatics investments. But the overall effort is slowed by the difficulty in obtaining quality samples in remote locations, transporting these to sequencing centers without degradation and producing the high molecular weight DNA (e.g., [1]) needed for long reads. Ethical and legal concerns also complicate sample collection and material transfer between countries. These limitations have restricted the growth of genomics in under-resourced communities. An ideal solution might be one where samples can be collected, processed, sequenced and assembled locally. In this study, we demonstrate that a portable sequencer, a relatively modest investment in equipment, and the availability of new software and communication tools can produce high-quality genome assemblies in a field-simulated setting.

## 2. Materials and Methods

### 2.1. Equipment and Supplies

We used a 6 M class B recreational vehicle (2023 Coachmen Nova C, Forest River Inc., Middlebury, IN, USA) equipped with refrigeration, microwave, Starlink (SpaceX Starlink, Hawthorne, CA, USA) satellite connectivity, Li battery, solar panel and 30 amp shore electrical availability as a field laboratory. Samples were processed when connected to grid power. A Bento Lab (bento.bio, London, UK) with gel box, thermal cycler and centrifuge for 1.5 or 2 mL tubes was used for DNA isolation, and library processing. DNA was isolated with either the New England Biolabs (NEB, Ipswich, MA, USA) Monarch High Molecular Weight DNA extraction kit for tissue (T3060) or blood (T3010) kits. Additional equipment included a small programmable dry temperature block (JOANLAB, Huzhou, China), a fluorimeter (Qubit, Life Technolgoies Holding, Singapore), various pipettors (Rainin Instruments; Oakland, CA, USA), an Oxford Nanopore Technologies (ONT, Oxford, UK) P2 solo sequencer, ONT LSK-114 ligation library kits, PromethIon R10.4.1 flow cells, a 2023 MacBook Pro M3 with 8 Tb of internal memory (Apple, Cupertino, CA, USA) and several 4 Tb SSDs (Samsung, Suwon, Republic of Korea) for data collection and backup.

### 2.2. Sample Source and Sequencing

The specimen was collected as roadkill at the approximate location of 36.435145, −115.371807 in southern Nevada and is exempt from permit requirements. It was estimated to be dead for less than 2 h when a 15 cm tail sample was taken for DNA isolation. A first isolation from a portion of the tail was done in the field, and the remaining tissue was frozen. Subsequent isolations were prepared from the remaining tissue in Baltimore as needed to achieve the required read depth. The ONT ligation kit LSK114 was used for library preparation, and the library was sequenced on PromethIon flow cells (R10.4.1) run on the P2 solo sequencer using an M3 MacBook Pro running MinKNOW (v24.11) to collect both the nucleotide and 5 mC/5 hmC methylation data. Run times were typically 72 h with washing and reloading at 24 h intervals, varying based on the performance of the particular library and flow cell, with 1–2 μg of DNA used per library without sizing or shearing. Four DNA libraries were made and run with the goal of acquiring at least 50× read depth as recommended for Hifiasm assembly. To simplify library preparation, several reagents were pipetted in the home laboratory into 0.2 mL tubes so that the sample could be transferred serially and quickly during the protocol. Pre-aliquoting reagents also allowed quick replacement of a tube in the event one was dropped or lost. Rather than the ONT-recommended 1.5 mL tubes, 0.2 mL tubes were used throughout the library prep because they were compatible with the Bento lab thermal block.

### 2.3. Assembly, Annotation and Analysis

Sequence was basecalled in Dorado (github.com/nanoporetech/dorado; version 1.1.1) as either high accuracy calling (HAC) or super-accurate calling (SUP). As HAC calling is faster, it was used locally, with 5 mC and 5 hmC enabled, but POD5 files were retained and were reprocessed subsequently on our cluster as SUP to produce unaligned BAMs (to retain methylation status). To maximize read quality, we used SUP calls for the de novo Hifiasm assembly and subsequent analyses. From four flow cells, we obtained a final combined read depth of 67× and an N50 of 18.3 kb.

The genome assembly was performed with Hifiasm-ONT (Hifiasm-0.25.0-r726; [2]), and a small number of remaining scaffolds were joined using RagTag (version 2.1.0; [3]) with the congeneric species *Masticophis lateralis* (NCBI GCA_030761175.1) used as the reference. Chromosomes were ordered by scaffold length with seqkit (2.10.1; [4]) relative to *Elaphe schrenckii* (Amur rat snake; NCBI GCA_050231175.1), a recent PacBio-generated chromosome assembly. The D-Genies (v.1.5.0; [5]) dot plot tool was used to visualize the chromosomal synteny. Annotation was performed using LiftOn [6] with the well annotated *Thamnophus sirtalis* genome (Western garter snake; NCBI GCA_009769535.1) as the source. BUSCO analysis (ver. 5.8.2; squamata_odb12; [7]) was used to assess completeness. A Snail plot [8] was generated to summarize assembly quality.

Mitochondrial sequences were identified with MitoFinder [9], assembled with MitoHiFi [10] as implemented on Galaxy version 3.2.3 [11] using *Elaphe bimaculata* (NC_024743.1; [12]) as the reference and annotation source. All thirteen expected protein-coding genes were found. Multiple reference sequences were chosen for use with different analysis tools, as no single closely related species had chromosome-level and mitochondrial assemblies with detailed annotations for both. More recent submissions that used long-read methods were prioritized.

## 3. Results

**Figure 1:**
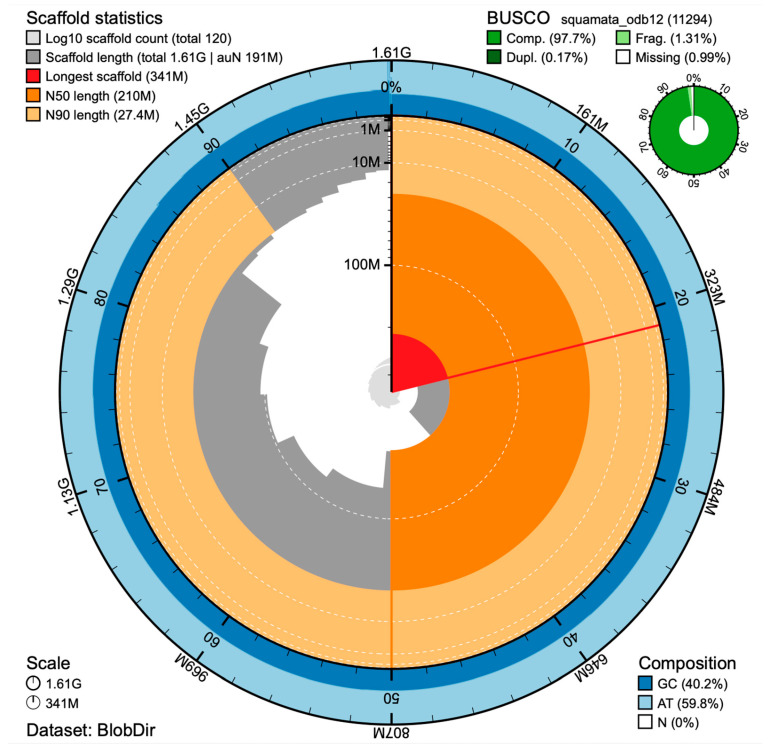
Snail plot [8] of scaffold statistics and BUSCO scores. The Snail plot illustrates that half the scaffolds have an N50 equal to or greater than 210 Mb, with the longest chromosome 341 Mb. The log10 scaffold count shows that all the sequences are accounted for in 120 scaffolds, which is significantly better than most current assemblies. The Busco plot illustrates the high number of complete protein-coding genes relative to fragmented or missing genes using squamata_odb12.

The field-isolated DNA provided the longest reads but lacked sufficient depth for analysis. Subsequent DNA isolations were performed, and when combined, had an N50 of 18.3 kb and a read depth of 67×. The Hifiasm-ONT assembly initially produced 152 scaffolds, which were reduced to 120 following RagTag joining using the *M. lateralis* reference. Of these, 18 were assigned to chromosomes (Table 1), and 102 were unplaced. The unplaced scaffolds totaled 3.116 Mb and averaged 30.55 kb, ranging from 694,998 to 1449 bp, representing 0.193 percent of all reads. Colubrid snakes have been reported to have 8 macrochromosomes and 10 microchromosomes [13], which agrees with our observation. The resulting nuclear genome was 1.61 Gb and had a BUSCO completeness score of 97.7% (S:11,015; D:19; F:148; M:112) from 11,294 BUSCO groups searched using the squamata_odb12 lineage dataset (Figure 1). The BUSCO score is comparable to that of the *Candoia aspera* (viper boa; GCF_035149785.1) and better than most current snake genomes. LiftOn [6] identified 19,832 total loci with 18,025 classified as protein coding. No protein-coding genes were identified in the unplaced scaffolds.

By convention, the RagTag assembly of the 18 chromosomes was named MFP_1-18 based on their length (Table 1). MFP_4 was identified as the sex determining Z chromosome based on the presence of the *CTNNB1* gene. Heterozygosity of the gene indicated that the specimen was male [13]. Scaffold statistics and BUSCO scores are shown in Figure 1.

**Figure 2:**
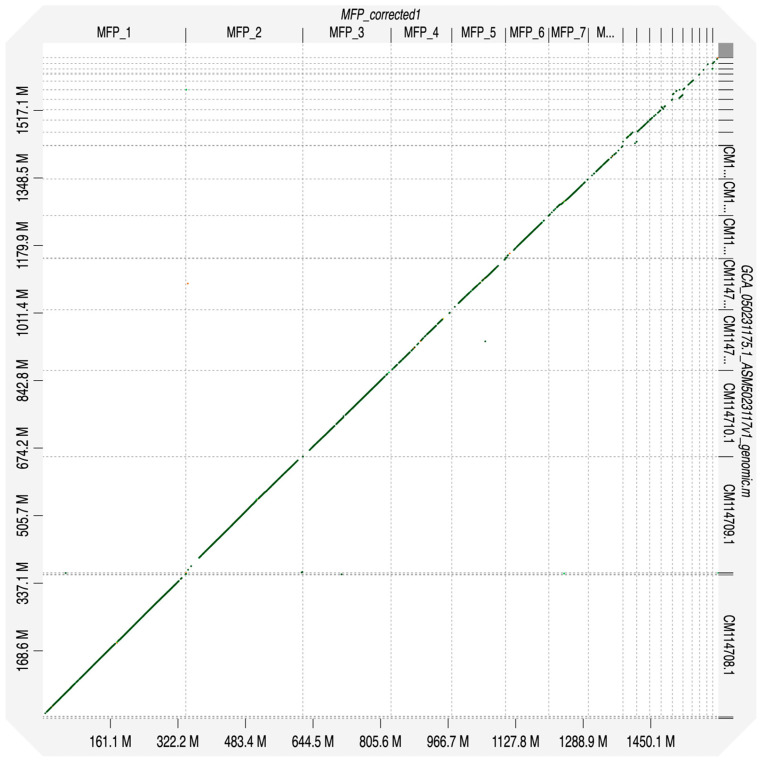
D-Genies [5] plot of the *M. flagellum piceus* assembly compared to *E. schrenckii* (Amur rat snake; [12]) assembled from PacBio reads with Hifiasm v. 024.0. The *M. flagellum piceus* scaffolds were ordered by size and reoriented by strand to best align with *E. schrenckii.* Unplaced scaffolds were excluded from the plot.

Figure 2 shows the D-Genies alignment of the *M. flagellum piceus* nuclear genome to *E. schrenckii*, chosen for comparison based on the quality of its assembly. The corresponding chromosomes (right and top axes) from each species show high concordance based on visual inspection. It is not possible to determine if small mismatches are the result of errors in either genome or true species differences.

We annotated the genome using tools that perform sequence comparisons, namely LiftOn [6], primarily for protein-coding genes and Earl Grey [14] for transposable element families. These tools are not replacements for the more thorough annotation processes used at NCBI or EBI, which include RNA sequence alignment, but as more genomes from related species become available, they are a reasonable proxy until a more formal annotation can be completed. Table 2 shows the major features identified by LiftOn, and Table 3 lists the transposable elements identified with Earl Grey.

### Mitochondrial Genome

**Figure 3:**
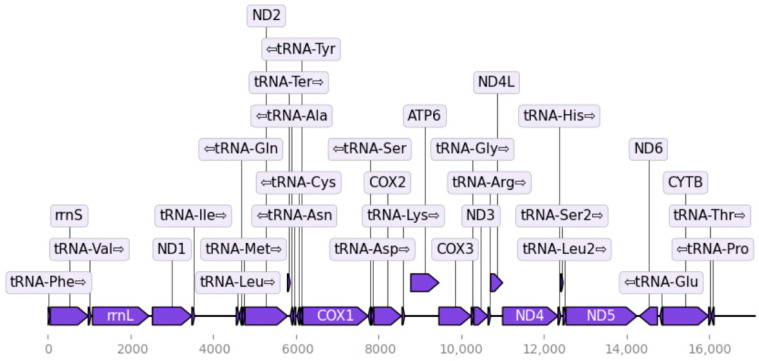
Mitofinder [9] and MitoHiFi ([10]; Galaxy v.3.2.3) produced a 17 kb assembly and map using *E. bimaculata* (NC_024743.1) as the reference and annotation source.

The mitochondrial genome produced with MitoHiFi was 17.12 kb and was predicted to include 13 protein-coding genes, 22 tRNAs and two rRNAs (Figure 3), as expected from other colubrid snakes. We found that the mitochondrial genome was 89.15% similar to *Coluber constrictor* (NC_ 071936.1) and 82.98% similar to *Elaphe bimaculata* using the NCBI BLAST tool (ver 2.17.0; https://blast.ncbi.nlm.nih.gov/).

## 4. Discussion

The nuclear and mitochondrial genomes were generated solely with nanopore sequencing on a portable sequencer and a laptop computer in field-simulated conditions. Subsequent analyses were done either using Galaxy [11] tools or at an institutional datacenter. The principal bioinformatic advances were the use of Hifiasm-ONT (ver 0.25.0-r726) to error correct and assemble nanopore reads and RagTag to combine the 32 remaining scaffolds, 3 of which were added to the Hifiasm-generated chromosomes. The quality of the genome alignment compared with that of the other snakes, the high BUSCO scores for the nuclear genome and the identification of the expected number of mitochondrial genes show that genomes can be assembled at relatively low cost and without the need for a large laboratory. We noted that the longest reads were obtained from the field-isolated sample rather than subsequent preparations from the frozen tissue. This is consistent with other studies (e.g, [1]) and with other projects we have done, where freshly prepared HMW DNA produced the longest reads. The primary current limitations with field-based sequencing are the speed of the ONT Dorado base calling and the rate of transferring data from the field to a datacenter or internet resources for subsequent analyses. New GPU devices and improved software are expected to do speed basecalling, satellite communication transfer speeds should continue to increase, and software tools that can run on higher-end laptops or online, such as Galaxy [11], will also continue to be refined. A vision of field-based genomics, especially in remote locations, that can be widely implemented globally and democratize genomics [15] is near realization.

## Figures and Tables

**Table 1:** Assembled chromosomes and lengths for *M. flagellum piceus*.

Chromosome	Length
MFP_1	340,894,829
MFP_2	279,161,145
MFP_3	210,491,607
MFP_4 (Z)	144,931,626
MFP_5	128,254,115
MFP_6	103,127,093
MFP_7	94,359,483
MFP_8	82,559,839
MFP_9	32,758,521
MFP_10	30,834,001
MFP_11	27,361,241
MFP_12	27,119,116
MFP_13	25,018,585
MFP_14	21,912,515
MFP_15	17,740,850
MFP_16	17,320,700
MFP_17	13,987,221
MFP_18	13,352,296

**Table 2:** Summary of LiftON [6] predicted gene annotations based on comparison to those of *T. sirtalis*.

Total features in reference:	21,085
Lifted features:	19,832
Protein-coding features:	18,025
Non-coding features:	1206
Other features:	601
Missed features:	1253
Total features in target:	21,764 (19,916 + 1206 + 642)
Protein-coding features:	19,916 (16452 + 3464)
single copy:	16,452
>1 copy:	3464 in total
Non-coding features:	1206 (1206 + 0)
single copy:	1206
>1 copy:	0, 0 in total
Other features:	642 (575 + 67)

**Table 3:** Transposable elements identified using Earl Grey v6.3.5 [14] in the 18 chromosomal assemblies.

TE Classification	Coverage (bp)	Copy Number	% Genome Coverage	TE Family Count
DNA	18,435,299	54,164	1.14200	54,164
Rolling Circle	782,719	4274	0.04849	4274
Penelope	21,220,964	111,150	1.31456	111,150
LINE	362,673,698	1,410,069	22.46630	1,410,062
SINE	36,366,789	216,235	2.25279	216,234
LTR	70,660,262	135,058	4.37714	135,058
Other (Simple Repeat, Microsatellite, RNA)	55,620,536	664,525	3.44549	664,524
Unclassified	280,506,098	1,305,488	17.37632	1,305,485
Non-Repeat	768,034,560	NA	47.57691	NA
Genome Size = 1,614,300,925				

## Data Availability

The genome has been deposited at NCBI under project number PRJNA1306602.

## References

[1] Dahn H.A., Mountcastle J., Balacco J., Winkler S., Bista I., Schmitt A.D., Pettersson O.V., Formenti G., Oliver K., Smith M. (2022). Benchmarking ultra-high molecular weight DNA preservation methods for long-read and long-range sequencing. Gigascience.

[2] Cheng H., Qu H., McKenzie S., Lawrence K.R., Windsor R., Vella M., Park P.J., Li H. (2025). Efficient near telomere-to-telomere assembly of Nanopore Simplex reads. bioRxiv.

[3] Alonge M., Soyk S., Ramakrishnan S., Wang X., Goodwin S., Sedlazeck F.J., Lippman Z.B., Schatz M.C. (2019). RaGOO: Fast and accurate reference-guided scaffolding of draft genomes. Genome Biol..

[4] Shen W., Sipos B., Zhao L. (2024). SeqKit2: A Swiss army knife for sequence and alignment processing. Imeta.

[5] Cabanettes F., Klopp C. (2018). D-GENIES: Dot plot large genomes in an interactive, efficient and simple way. PeerJ.

[6] Chao K.-H., Heinz J.M., Hoh C., Mao A., Shumate A., Pertea M., Salzberg S.L. (2025). Combining DNA and protein alignments to improve genome annotation with LiftOn. Genome Res..

[7] Simão F.A., Waterhouse R.M., Ioannidis P., Kriventseva E.V., Zdobnov E.M. (2015). BUSCO: Assessing genome assembly and annotation completeness with single-copy orthologs. Bioinformatics.

[8] Challis R.J., Blaxter M.L. (2025). Snail plots are badges of genome assembly quality. bioRxiv.

[9] Allio R., Schomaker-Bastos A., Romiguier J., Prosdocimi F., Nabholz B., Delsuc F. (2020). MitoFinder: Efficient automated large-scale extraction of mitogenomic data in target enrichment phylogenomics. Mol. Ecol. Resour..

[10] Uliano-Silva M., Ferreira J.G.R.N., Krasheninnikova K., Formenti G., Abueg L., Torrance J., Myers E.W., Durbin R., Blaxter M., Darwin Tree of Life Consortium (2023). MitoHiFi: A python pipeline for mitochondrial genome assembly from PacBio high fidelity reads. BMC Bioinform..

[11] (2024). The Galaxy Community, The Galaxy platform for accessible, reproducible, and collaborative data analyses: 2024 update. Nucleic Acids Res..

[12] Fan J., Huang R., Yang D., Gong Y., Cui Z., Wang X., Su Z., Yu J., Zhang Y., Zhang T. (2023). The Genome Assembly of the King Ratsnake Elaphe carinata, Helps Reveal Its Biological Characteristics. Gigabyte.

[13] Viana P.F., Ezaz T., de Bello Cioffi M., Jackson Almeida B., Feldberg E. (2019). Evolutionary Insights of the ZW Sex Chromosomesin Snakes: A New Chapter Added by the AmazonianPuffing Snakes of the Genus Spilotes. Genes.

[14] Baril T., Galbraith J., Hayward A. (2024). Earl Grey: A Fully Automated User-Friendly Transposable Element Annotation and Analysis Pipeline. Mol. Biol. Evol..

[15] Blaxter M., Lewin H.A., DiPalma F., Challis R., da Silva M., Durbin R., Formenti G., Franz N., Guigo R., Harrison P.W. (2025). The Earth BioGenome Project Phase II: Illuminating the eukaryotic tree of life. Front. Sci..

